# Transplantation of Human Amniotic Membrane over the Liver Surface Reduces Hepatic Fibrosis in a Cholestatic Model in Young Rats

**DOI:** 10.1155/2018/6169546

**Published:** 2018-02-25

**Authors:** M. Garrido, C. Escobar, C. Zamora, C. Rejas, J. Varas, C. Córdova, C. Papuzinski, M. Párraga, S. San Martín, S. Montedonico

**Affiliations:** ^1^Centro de Investigaciones Biomédicas, Universidad de Valparaíso, Valparaíso, Chile; ^2^Servicio de Cirugía Pediátrica, Hospital Carlos van Buren, Valparaíso, Chile; ^3^Departamento de Salud Pública, Universidad de Valparaíso, Valparaíso, Chile

## Abstract

**Purpose:**

Biliary atresia precedes liver cirrhosis and liver transplantation. Amniotic membrane (AM) promotes tissue regeneration, inhibits fibrosis, and reduces inflammation. Here, we test amniotic membrane potential as a therapeutic tool against cholestatic liver fibrosis.

**Methods:**

Three groups of rats were used: sham surgery (SS), bile duct ligature (BDL), and bile duct ligature plus human amniotic membrane (BDL + AM). After surgery, animals were sacrificed at different weeks. Biochemical and histopathological analyses of liver tissue were performed. Collagen was expressed as a percentage of total liver tissue area. qPCR was performed to analyse gene expression levels of transforming growth factor-*β*1 (*Tgfb1*) and apelin (*Apln*). Statistical analysis performed considered *p* < 0.05 was significant.

**Results:**

Groups undergoing BDL developed cholestasis. Biochemical markers from BDL + AM group improved compared to BDL group. Ductular reaction, portal fibrosis, and bile plugs were markedly reduced in the BDL + AM group compared to BDL group. Collagen area in BDL + AM group was statistically decreased compared to BDL group. Finally, expression levels of both *Apln* and *Tgfb1* mRNA were statistically downregulated in BDL + AM group versus BDL group.

**Conclusion:**

AM significantly reduces liver fibrosis in a surgical animal model of cholestasis. Our results suggest that AM may be useful as a therapeutic tool in liver cirrhosis.

## 1. Background and Purpose

Biliary atresia is a neonatal disease of unknown etiology characterized by progressive, inflammatory, and fibrosclerosing cholangiopathy resulting in obstruction of both extrahepatic and intrahepatic bile ducts. Despite Kasai portoenterostomy, 68–80% of patients affected by biliary atresia develop progressive fibrosis leading to biliary cirrhosis and portal hypertension [1]. Biliary atresia constitutes the first cause of pediatric liver transplantation, accounting for half of cases [2]. In the last two decades, strategies to improve liver function in biliary atresia have been focused in early diagnosis and in developing methods to prevent progressive liver fibrosis.

Animal models play a key role in our understanding of the molecular basis and natural history of human diseases. Bile duct ligature (BDL) is a surgical model of cholestasis that provokes a progressive liver fibrosis leading to liver cirrhosis [3–5].

In the least years, stem cells have been successfully used for regenerative therapies against human diseases. The amniotic membrane retains pluripotential properties from epiblastic cells offering anti-inflammatory and antifibrotic properties; it modulates angiogenesis and favours healing process. Amniotic membrane has unlimited availability and has not ethical or legal barriers. Preclinical studies have demonstrated that amniotic membrane reduces lung and liver fibrosis in animal models, but only adult animals have been tested [6–11].

The aim of this study was to test human amniotic membrane as a potential therapeutic tool against cholestatic liver fibrosis in young rats.

## 2. Methods

### 2.1. Animals

The study was approved by the Institutional Bioethics Committee for Animal Research of Universidad de Valparaíso (BEA029-2014). It was carried out according to the International Guiding Principles regarding the use of laboratory animals [12].

Sixty-three 3-week-old Sprague-Dawley rats were divided into three groups: sham surgery (SS, *n* = 21), bile duct ligature (BDL, *n* = 19), and bile duct ligature plus human amniotic membrane (BDL + AM, *n* = 20). Numbers are different because two rats from the BDL group and one rat from the BDL + AM group died after surgery and were not analyzed. They had access to drink and food ad libitum, circadian cycle of light-darkness 12 : 12 h, controlled humidity (40–70%), and temperature (21 ± 2°C), with ventilation of 10 changes air/h.

### 2.2. Donor Selection and Procurement of Amniotic Membrane

Full-term placentas were collected from caesarean section delivery of healthy women donor under informed consent. Institutional Ethic Committee approved the protocol of placenta and amniotic membrane donation (88-04.09.2013).

Placenta and amniotic membrane were processed under sterile conditions; in a laminar flow hood, blood clots were removed with saline solution 0.09% plus penicillin [50 *μ*g/mL], streptomycin [50 *μ*g/mL], and amphotericin B [2.5 *μ*g/mL]. Amniotic membrane was isolated from chorion through blind dissection. These samples were cultured in order to rule out bacterial or fungal infection. If cultures were negative, amniotic membranes were frozen in sterile Petri dish with Dulbecco's modified Eagle medium at −80°C and glycerol in 1 : 1 proportion.

### 2.3. Surgical Procedure

**Figure 1:**
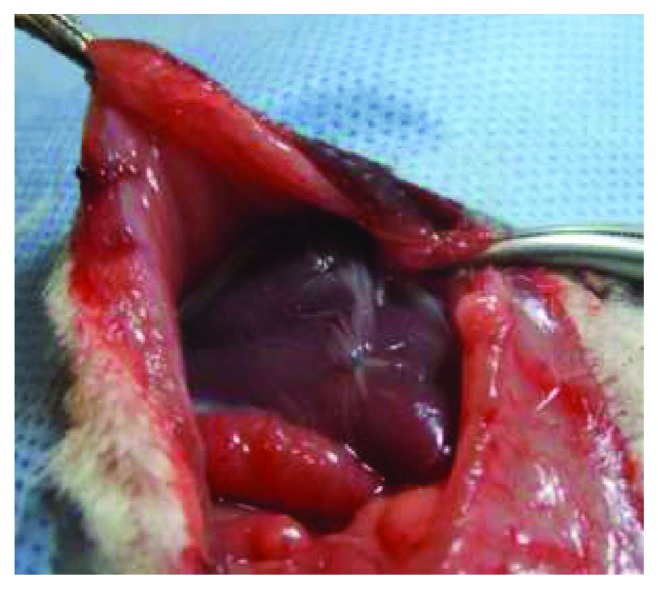
After the completion of bile duct ligature procedure, in the BDL + AM group, human amniotic membrane was sutured to the hepatic surface covering the whole liver.

One hour before the operation, acetaminophen 0.1 mL (10 g/100 mL) was administered to 3-week-old rats by oral route. The rat was placed in a transparent acrylic box for anesthetic induction. The rat was preoxygenated with 99.5 ± 0.5 pure oxygen at 1 L/min for 30 s and then anesthetized with isoflurane at 3-4% with a funnel-fill vaporizer (Harvard Apparatus, Holliston, MA, USA) until assessed by the absence of voluntary movements, muscle relaxation, and loss of response to stimuli reflexes. After that, the rat was placed in a heating small-animal operating table (Harvard Apparatus, Holliston, MA, USA). Anesthesia was maintained with a facial mask with oxygen and isoflurane 2%. The surgical procedure was performed under clean conditions, using surgical loupes (magnification 2.5x) and microsurgical instruments. A superior midline abdominal incision was performed. The viscera were exposed, the duodenum was exteriorized, and the common bile duct was identified and dissected. After that, the common bile duct was ligated into two parts: a distal ligature was placed just before the entrance to the pancreas, and a proximal ligature was placed below the hepatic duct junction, both with 7-0 polypropylene. After that, the common bile duct was sectioned in the middle. In the BDL + AM group, human amniotic membrane was sutured to the hepatic surface with 7-0 polypropylene covering the whole liver (Figure 1). The abdomen was then closed in two layers. In the SS group, the common bile duct was dissected, but it was not ligated. During anesthetic recovery, the rats were exposed to infrared light to prevent hypothermia for 30 min and received oxygen until awake. After surgery, the rats were allowed to feed normally. After the surgical procedure, rats were followed up on a daily basis until their sacrifice.

### 2.4. Euthanasia and Collection of Samples

Rats from each group were sacrificed in subgroups of 7 on the 2nd, 4th, and 6th postoperative weeks, under general anesthesia with isoflurane 4% according to the International Guiding Principles regarding the use of laboratory animals [12]. Weight gain (grams) was recorded before euthanized. A laparotomy was performed describing the intra-abdominal anatomy focusing in liver aspect and biliary duct changes.

### 2.5. Biochemical Liver Function Analysis

Blood samples were collected by puncture with a 21-gauge needle from the infrahepatic inferior cava vein at time of euthanasia. These were centrifuged at 1000 rpm by 3 min and analyzed by VetTest® Chemistry Analyzer (IDEXX Laboratories, Westbrook, ME, USA). Albumin, alkaline phosphatase, alanine transaminase, gamma-glutamyl transpeptidase, and total bilirubin were studied.

### 2.6. Determination of Spleen Index

The spleen was extracted and weighed (grams) in order to obtain an indirect measurement of portal hypertension expressed as “spleen index”: spleen weight/body weight × 100 [3].

### 2.7. Histopathological Examination

The left lateral lobe of the liver from each rat was subject to histological evaluation. A sample of the lobe was divided into two samples: the first sample was kept at 4% buffered paraformaldehyde, embedded in paraffin, sectioned, and stained with hematoxylin-eosin. The second sample was fixed in methacarn solution (methanol, chloroform, and acetic acid: 6 : 3 : 1) during 4 h at 4°C. After that, samples were dehydrated by submerging into 100% ethanol 3 times for 30 minutes each, cleared in xylol 3 times for 15 minutes each, and embedded in liquid paraffin at 60°C 3 times for 30 minutes each, and finally included in a solid paraffin block. From these blocks, 10 histological sections of 5 *μ*m thick were cut, and number 11 was the selected section for analysis. Another 10 sections of 5 *μ*m were cut, and number 11 was again selected for analysis. At the end, two sections, 5 *μ*m each, separated by 50 *μ*m of tissue were analyzed. This makes a total number of 42 sections from group SS, 38 sections from group BDL, and 40 sections from group BDL + AM. This procedure was carried out with every liver. Selected sections were stained in a sirius red solution during 1 h for collagen evaluation. The slides were analyzed in a light microscope (Olympus® CX81, Olympus, Tokyo, Japan) and photographed (DP71 camera, Olympus, Tokyo, Japan). Images at 40x, 100x, and 400x magnification were captured.

### 2.8. Collagen Quantification

Ten nonoverlapping photographs from random fields for each selected section as described above were taken from two slides per specimen at 100x magnification. We obtained 42 × 10 = 420 images from group SS, 38 × 10 = 380 images from group BDL, and 40 × 10 = 400 images from group BDL + AM. Images were exported in .JPG format with a resolution of 1200 × 900 pixels in RGB color palette. They were then converted in TIFF format and processed to enhance the positive marking, adjusted using the hue-saturation-brightness (HSB) combination with a graphic editor (Adobe Photoshop CC version 16.1.0, Adobe Systems, San José, CA, USA). Images were processed in an automated image analyzer (ImageJ version 1.48, US National Institutes of Health, Bethesda, MA, USA). Threshold colors were adjusted using HSB combination to select all the positive sirius red marking according to controls. Selected area was measured in square pixels, and the staining intensity was measured in an 8-bit gray scale (0 to 256). The positive area fraction considering a complete picture of the same resolution was calculated. The process of analysis was automated using macrocode to process 10 images per cycle. Data were expressed as a percentage of total liver tissue area.

### 2.9. Gene Expression

A sample of 100 mg from left lateral lobe of the liver was frozen in liquid nitrogen and used for gene expression analysis. Total RNA was extracted using TRIzol® RNA Isolation Reagent (Ambion, Thermo Fisher Scientific, Waltham, MA, USA) according to the manufacturer's recommendations. Template cDNA was obtained by reverse transcription of 2 *μ*g of total RNA using MMLV retrotranscriptase (NEB, Ipswich, MA, USA). Reaction mixtures were incubated at 25°C for 10 min, 42°C for 50 min, and 70°C for 10 min.

Relative quantification of gene expression levels for transforming growth factor-*β*1 (*Tgfb1*) and apelin (*Apln*) genes was carried out by real-time quantitative PCR (RT-PCR) on EcoPCR real-time system (Illumina, San Diego, CA, USA) using cDNA samples obtained as described before. For this purpose, SYBR® Select Master Mix (Thermo Fisher Scientific, Waltham, MA, USA) was used according to manufacturer's instructions. Specific primers were designed for amplification of each gene, and their sequences are described below. Comparative cycle threshold (Ct) values were obtained after RT-PCR reaction was performed. We used the Ct method to calculate relative mRNA expression. All quantifications were normalized by the corresponding expression of *β*2 microglobulin (*B2m*) mRNA that served as housekeeping gene. In addition, results were corrected with the expression of the same normalized genes in control tissue. Finally, gene expression differences were calculated using data obtained from groups according to the time elapsed after surgery. These differences were shown as “fold change” in the level of expression of both *Apln* and *Tgfb1* at 2, 4, and 6 weeks after surgery.

The sequences of the primers used in this study were presented in Table 1.

### 2.10. Statistical Analysis

Data are expressed as mean ± standard deviation. ANOVA test was used for comparison of initial weights between three groups. Nonparametric Mann–Whitney Rank Sum Test was applied to evaluate other differences among groups. A *p* value < 0.05 was considered statistically significant. Statistical analysis was performed using STATA software version 12.0 (StataCorp LP, College Station, TX, USA).

## 3. Results

**Figure 2:**
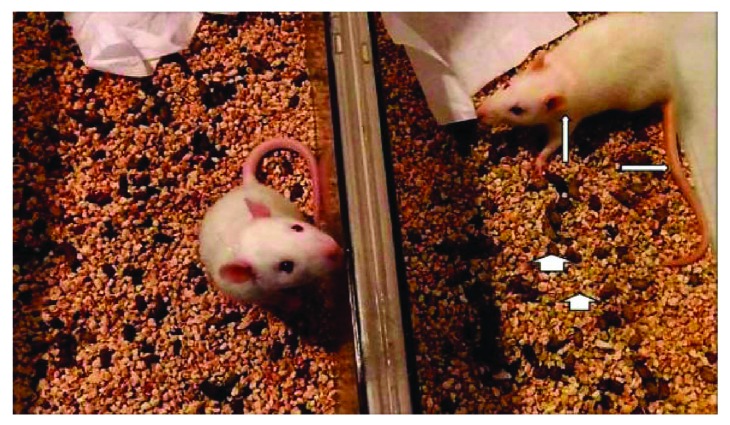
In comparison with the control group, in both the BDL groups, cholestasis was clinically evident, characterized by icteric coloration of face, legs, and tail (arrows). Additionally, yellowish pigmentation was observed in the gravel of boxes (choluria), and stools were clearly pale (acholia) (arrowheads).

**Figure 3:**
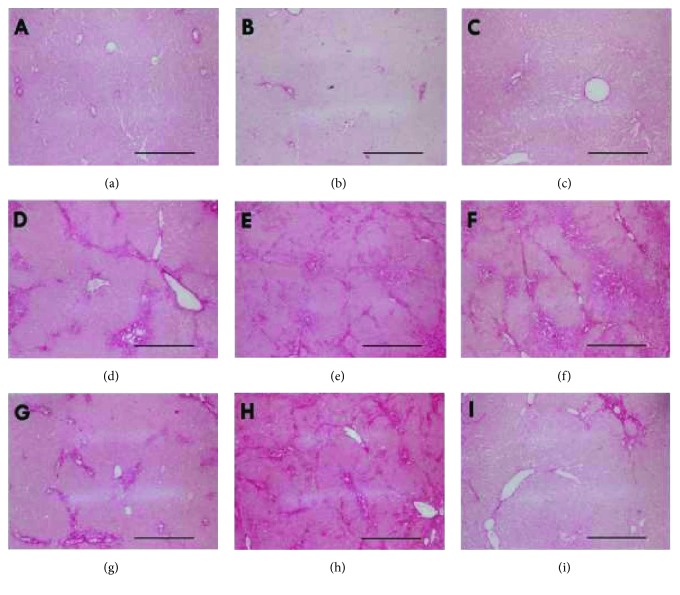
Representative histological sections stained with sirius red from the three studied groups on the 2nd, 4th, and 6th postoperative weeks. In the SS group, no changes were noticed (a–c). In the BDL group, a progressive ductular reaction and periportal fibrosis were evident along the postoperative weeks (d–f). In the BDL + AM group, a progressive ductular reaction and periportal fibrosis were noted on the 2nd and 4th postoperative weeks (g and h). However, a marked reduction in liver fibrosis was evident on the 6th postoperative week (i). SS: sham surgery; BDL: bile duct ligature; BDL + AM: bile duct ligature plus amniotic membrane; PO: postoperative. Scale bar represents 200 *μ*m.

**Figure 4:**
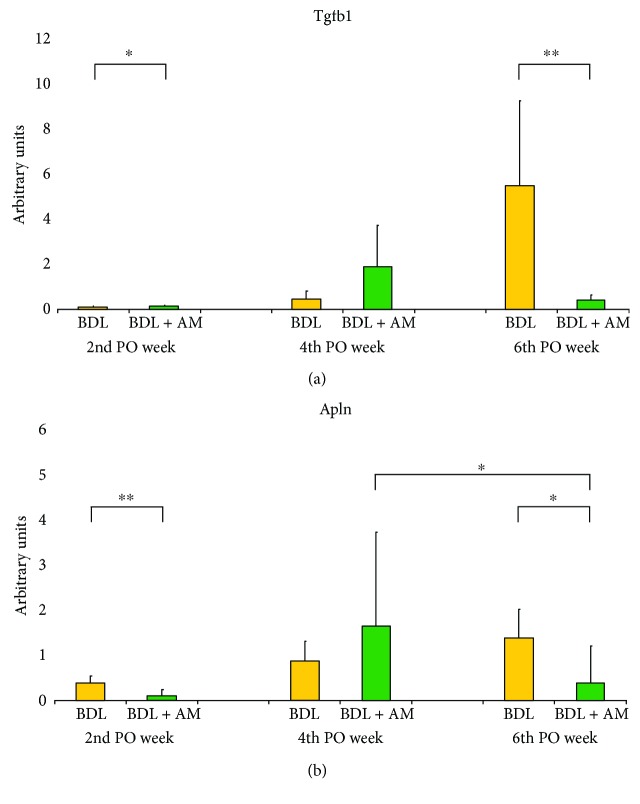
mRNA expression levels of profibrogenic genes in arbitrary units as “fold change.” (a) mRNA expression levels of *Tgfb1*. In the BDL group, *Tgfb1* exponentially increased along the experiment. Marked differences were noted on the 6th postoperative week between the BDL group and the BDL + AM group. (b) mRNA expression levels of *Apln*. In the BDL group, *Apln* increased in a linear way over time. Marked differences were also noticed on the 6th postoperative week between the BDL group and the BDL + AM group. Additionally, a reduction in expression levels of *Apln* was noticed in the BDL + AM group among the 4th and 6th postoperative weeks. SS: sham surgery; BDL: bile duct ligature; BDL + AM: bile duct ligature plus amniotic membrane; PO: postoperative. ^∗^*p* < 0.05 BDL versus BDL + AM; ^∗∗^*p* < 0.01 BDL versus BDL + AM.

All the rats in the SS group survived the experiments, while the survival rate in the BDL group was 19/21 and in the BDL + AM group was 20/21. In both the BDL and BDL + AM groups, cystic dilatation of the bile duct was observed in all the rats studied and was a consequence of the surgical procedure of bile duct ligation model, demonstrating that the bile duct was effectively obstructed. Cholestasis was clinically evident and progressive in time, noted by icteric coloration of face, legs, and tail. Additionally, yellowish pigmentation was observed in the gravel of boxes (choluria), and stools were clearly pale (acholia) (Figure 2). At laparotomy, bile-stained tissues, ascites, and splenomegaly was observed in all the rats studied. All these findings were progressive in time and were more striking in the rats that were sacrificed on the 6th postoperative week. In addition, poor growth of hair in the surgical wound was observed.  Table 2 shows preoperative and postoperative weight and spleen index in rats. No initial or postoperative differences were noted in the three groups. In both the BDL and BDL + AM groups, histological analysis of liver showed progressive ductular reaction and portal fibrosis with collagen deposition. However, on the 6th postoperative week, the BDL + AM group presented a marked reduction in these features (Figure 3 and Table 3). Biochemical liver function analysis is shown in Table 4. mRNA expression level of *Tgfb1* and *Apln* was significantly higher in the BDL group versus the BDL + AM group, with values for *Tgfb1* of 5.49 ± 3.7 versus 0.30 ± 0.25, respectively (*p* < 0.01), and values for *Apln* of 1.40 ± 0.6 versus 0.39 ± 0.8, respectively (*p* < 0.05) (Figure 4).

## 4. Discussion

Biliary atresia constitutes an important problem in pediatric surgery. The vast majority of patients would need a liver transplantation. Children undergoing liver transplantation for biliary atresia are younger than those engrafted for other conditions displaying a higher risk of complications and retransplantation [2]. Therefore, studying strategies to improve liver function secondary to biliary atresia is mandatory. Three types of animal models have been reported in biliary atresia: spontaneous, secondary to viral infection, and surgically created. The sea lamprey (*Petromyzon marinus*) is a jawless vertebrate that during metamorphosis from larvae to parasitic juveniles progressively loses the biliary system until complete biliary degeneration. However, sea lamprey does not develop cirrhosis during development of biliary atresia. Intraperitoneal inoculation of mice with rhesus rotavirus within the first 48 h of postnatal life leads to injury of the biliary epithelium and subsequent extrahepatic biliary obstruction and intrahepatic bile duct proliferation. However, rhesus rotavirus model is limited by timing and dosing of virus application, injection-related injury to abdominal organs, cannibalization, and survival rate of pups. In addition, mice do not develop liver fibrosis and portal hypertension because they recover spontaneously [13]. Bile duct ligature (BDL) is a surgically created animal model developed by Cameron and Oakley in 1932 that consists in ligation and excision of the common bile duct, being the most utilized to replicate cholestasis. BDL is the only animal model that allows progressive liver damage with long-term follow-up and recreates associated complications such as portal hypertension and hepatopulmonary syndrome. Gibelli et al. demonstrated that histological and molecular findings in livers of newborn rats submitted to BDL were more similar to biliary atresia-related fibrosis pattern compared to adult rats [5]. However, operating on in rat pups is technically difficult because of the small size of biliary structures and mortality after the procedure was reported to be as high as 77% [3–5]. In the present study, we developed BDL in 3-week-old rats. At that age, ligature of the common bile duct is technically less complex to perform than in newborn rats, and animals can be kept without their mothers during the postoperative period avoiding cannibalism. Our study demonstrates that BDL in 3-week-old rats recreates all the histological and molecular features of biliary atresia, having the advantages of both neonatal and adult rat models of bile duct ligature. Additionally, our study demonstrates that the survival rate of young rats is superior to previous reports due to appropriate perioperative care in anesthesia and recovery, by controlled anesthetic delivery and avoiding hypothermia.

Regenerative medicine is an emerging multidisciplinary field focused on replacing or regenerating human cells, tissues, or organs in order to restore or establish normal function. A few reports have explored the use of stem cells in liver failure after biliary atresia. Gupta et al. injected autologous bone marrow mononuclear stem cells into hepatic artery and/or portal vein in eight patients demonstrating an initial improve in liver function [14]. After that experience, the authors included fifteen children with biochemical and scintigraphic improvement at 1-year follow-up [15]. Other authors reported a 1-year-old girl treated with hepatic progenitor cell infusion through the hepatic artery with decreased in serum bilirubin values and a scintigraphy showing increased liver cell function after 2 months [16].

However, the use of stem cells has some drawbacks. Embryonic stem cells collection implies important ethical barriers. Embryonic stem cells also have tumorigenic capacity. Adult mesenchymal stem cells are present in lower concentration, require invasive procedures for collection, and have limited proliferation [17]. In contrast, amniotic membrane, the innermost layer of fetal membranes, has many advantages over other sources of stem cells. Placenta is a readily available organ, with minimum ethical and legal barriers. There is no need for invasive procedures, and amniotic membrane stem cells are no tumorigenic. Moreover, stem cells from amniotic membrane induce apoptosis in neoplastic cells [18]. Amniotic stem cells have more plasticity and do not have accumulative damage in DNA, supporting better time lapse in preservation conditions [10]. Other properties of amniotic membrane include barrier mechanism and analgesic effects, when used as a biological dressing, preventing desiccation and excessive fluid loss, protecting exposed sensitive nerve ends in wound bed from the environment. Low immunogenicity is another property of amniotic membrane. This is explained by lack of human leukocyte antigen (HLA) class A, B, and DR and costimulatory molecules CD40, CD80, and CD86 in human amniotic cells [19]. Amniotic membrane promotes epithelization by releasing of growth factors and retains basement membrane components that influence proliferation, migration, and differentiation of epithelial cells. Additionally, amniotic membrane has antibacterial properties by production of human beta-3-defensin, elastase inhibitors, secretion of leukocyte proteinase inhibitor, lactoferrin, and IL-1RA. Finally, amniotic membrane has a role in angiogenesis modulation. It produces pro-angiogenic compounds such as vascular endothelial growth factor (VEGF) and basic fibroblastic growth factor (bFGF). On the other hand, amniotic membrane produces anti-angiogenic factors as thrombospondin-1, IL-1RA, collagen XVIII, IL-10, some tissue metalloprotease inhibitors, and pigment epithelium-derived factor [19].

The use of amniotic membrane in hepatic disease models is emerging. A study demonstrated a reduction in pro-inflammatory cytokines IL-6 and TNF*α* after intravenous administration of human amniotic epithelial cells in a carbon tetrachloride toxic model [6]. Sant'Anna et al. demonstrated a reduction in liver fibrosis after amniotic membrane application on the liver surface in a bile duct ligature model; however, they used adult rats [8, 11]. In the present study, we demonstrated a reduction in hepatic fibrosis in young rats by liver function analysis, by collagen quantification, and by analysis of profibrotic genes.

Liver diseases are characterized by a catabolic state compromising nutrition. In our study, weight gain was recorded in the three groups along the experiments. At the end of study, no significant differences were noted among the three groups. These findings may be due to compensatory weight gain in both the BDL and BDL + AM groups by development of hepatosplenomegaly and ascites as a consequence of a progressive liver fibrosis and portal hypertension. Spleen index is a ratio between spleen weight and body weight that allows to study the presence of splenomegaly and secondary portal hypertension [3]. In our study, both the BDL and BDL + AM groups developed progressive increase in spleen index, without differences among them. Previous studies using amniotic membrane in liver diseases have not measured the spleen index [6–11].

Adult mice treated with injection of human amniotic epithelial cells showed lower levels of alanine transaminase after injection [6]. Similarly, our results show an improvement in liver function tests, but only albumin and gamma-glutamyl transpeptidase had a significant change most probably due to the limited number of samples studied.

Liver fibrogenesis is a dynamic wound healing-like process leading to progressive accumulation of extracellular matrix components. Histopathological liver changes in biliary atresia include ductular reaction, portal fibrosis, and bile plugs. Ductular reaction consists in proliferation of small interanastomosing ductules located at the periphery of portal tracts and represents the most consistent indicator of the presence of biliary obstruction [20]. Portal fibrosis pattern is characterized by formation of portal–portal fibrotic septa surrounding liver nodules [21]. Collagen quantification by digital image analysis allows a more precise and objective measurement of fibrosis avoiding interobserver variations [22]. All ductular reaction and portal fibrosis were found in rat livers after BDL procedure and were clearly progressive in time, as demonstrated by collagen quantification. After six weeks of treatment with amniotic membrane, a significant reduction of liver fibrosis was evident. Manuelpillai et al. injected human amniotic epithelial cells into a carbon tetrachloride model of liver fibrosis. They demonstrated a reduction in one third of hepatic fibrosis assessed by image analysis of sirius red stained slides [6]. Sant'Anna et al. applied amniotic membrane in a bile duct ligature model of liver fibrosis in adult rats [8]. They demonstrated a reduction of a half in collagen deposition in the treated group assessed by image analysis of Goldner's modified Masson trichrome stained slides [8]. Recently, then same authors demonstrated similar results in a bile duct ligature model of liver fibrosis in adult rats using sirius red staining. Our results show the same reduction in collagen deposition after amniotic membrane treatment in young rats.

In order to explain the potential mechanism involved in fibrosis reduction by human amniotic membrane, we explored the expression levels of two profibrotic genes: an established major profibrogenic cytokine, *Tgfb1*, and a novel involved protein in liver fibrosis recently linked to prognosis of biliary atresia patients, *Apln*.

TGF-*β* is a central regulator in chronic liver diseases that contributes to all stages of disease progression from initial liver injury through inflammation and fibrosis to cirrhosis and hepatocellular carcinoma. TGF-*β*1 isoform is considered as a major profibrogenic cytokine. TGF-*β*1 regulates a wide variety of cellular processes in liver fibrogenesis, including apoptosis of hepatocytes, enhance hepatocyte destruction, hepatic stellate cell and fibroblast activation with type I collagen production, recruitment of inflammatory cells into injured liver, and transdifferentiation of some liver-resident cells. In our study, *Tgfb1* gene expression had an exponential overexpression on the 6th postoperative week in the BDL group in accordance with the previous reports [23]. TGF-*β*1 inactivation reduces collagen synthesis, and this pathway is indicated as accountable for reversion of liver fibrosis by amniotic membrane. This effect over *Tgfb1* expression and type I collagen deposition has also been implied in playing a role in scarless fetal wound healing [19]. In addition, collagen deposition reduction was observed in a lung fibrosis model by induction of a collagen-degrading environment with altered proteases levels [7]. Our results show that the BDL + AM group *Tgfb1* gene expression was significantly downregulated on the 6th postoperative week demonstrating that amniotic membrane reverses collagen deposition by inhibiting *Tgfb1* gene expression in cholestatic young rats.

Apelin, the endogenous ligand of angiotensin-like-receptor 1, is an emergent peptide involved in liver disease. Chen et al. demonstrated that *apelin* is overexpressed in livers of biliary atresia patients according to progression of disease and that is markedly activated in end-stage cirrhosis [24]. The authors demonstrated that *apelin* expression level accurately reflects the severity of hepatic fibrosis and proposed that it could be used as a prognostic factor in biliary atresia patients, to estimate the timing of liver transplantation [24]. Our results show a linear progressive overexpression of *Apln* in the BDL group. In the BDL + AM group, *Apln* showed a downregulation in association to reversion of collagen deposition. This study is the first to evaluate *Apln* expression in a cholestatic model in young animals. Our results support the use of apelin as a prognostic factor in biliary atresia.

In conclusion, human amniotic membrane applied over liver surface reverses by near 50% liver fibrosis in a surgical model of cholestasis in young animals. These results suggest that amniotic membrane may be useful as a therapeutic tool against liver fibrosis in neonatal and childhood cholestatic disorders.

## Figures and Tables

**Table 1:** The sequences of the primers.

Gene	Sense primer	Antisense primer
*Tgfb1*	5′-AGAGCCCTGGATACCAACTA-3′	5′-GACCTTGCTGTACTGTGTGT-3′
*Apln*	5′-TGTCCTCATCCCGTGTGTTC-3′	5′-AAGCACTCACCTCCCTACA-3′
*B2m*	5′-CAGTTCCACCCACCTCAGAT-3′	5′-TTTTGGGCTCCTTCAGAGTG-3′

**Table 2:** Preoperative and postoperative weight and spleen index.

		Preoperative	2nd PO week	4th PO week	6th PO week
SS (*n* = 21)	Weight	52.65 ± 6.68 g	129.46 ± 12.97 g	213.74 ± 37.74 g	235.19 ± 37.17 g
	Spleen index		0.42 ± 0.03	0.31 ± 0.06	0.26 ± 0.05

BDL (*n* = 19)	Weight	54.16 ± 9.41 g	85.29 ± 38.90 g^∗^	154.37 ± 27.68 g^∗^	219.64 ± 47.99 g
	Spleen index		0.38 ± 0.34	0.60 ± 0.11	0.54 ± 0.24^∗^

BDL + AM (*n* = 20)	Weight	51.37 ± 12.95 g	62.08 ± 15.92 g^†^	116.74 ± 25.26 g^††‡^	241.30 ± 48.67 g
	Spleen index		0.38 ± 0.14	0.53 ± 0.15^∗∗^	0.54 ± 0.28^∗^

Weight is measured in grams (g), and spleen index in absolute values.

PO: postoperative; SS: sham surgery; BDL: bile duct ligature; AM: amniotic membrane.

^∗^ *p* < 0.05 sham versus BDL/BDL + AM.

^∗∗^ *p* < 0.01 sham versus BDL/BDL + AM.

^†^ *p* < 0.05 sham versus BDL + AM.

^††^ *p* < 0.01 sham versus BDL + AM.

^‡^ *p* < 0.01 BDL versus BDL + AM.

**Table 3:** Collagen quantification.

	2nd PO week	4th PO week	6th PO week
SS (*n* = 21)	4.81 ± 4.72%	3.05 ± 3.50%	4.80 ± 4.15%
BDL (*n* = 19)	14.25 ± 6.74%^∗∗^	16.29 ± 9.46%^∗∗^	26.96 ± 11.63%^∗∗^
BDL + AM (*n* = 20)	14.70 ± 6.80%^∗∗^	19.28 ± 9.33%^∗∗^	14.57 ± 9.37%^∗∗^^††‡^

PO: postoperative; SS: sham surgery; BDL: bile duct ligature; AM: amniotic membrane.

^∗∗^ *p* < 0.01 sham versus BDL/BDL + AM.

^††^ *p* < 0.01 BDL versus BDL + AM.

^‡^ *p* < 0.05 BDL + AM 4th PO week versus BDL + AM 6th PO week.

**Table 4:** Liver function tests.

	Sham (*n* = 6)	2nd PO week (*n* = 3)	4th PO week (*n* = 4)	6th PO week (*n* = 5)
Albumin (g/dL)	2.5 ± 0	BDL: 2.4 ± 0.37 BDL + AM: 2.85 ± 0.07	BDL: 1.88 ± 0.5^∗^ BDL + AM: 2.9 ± 0^**†**^	BDL: 1.99 ± 0.17 BDL + AM: 3.33 ± 0.36^**‡†**^

ALT (U/L)	70 ± 7.07	BDL: 79.75 ± 17.99 BDL + AM: 106 ± 0	BDL: 124.6 ± 58.8 BDL + AM: 76 ± 7.07	BDL: 79 ± 46.16 BDL + AM: 56.71 ± 35.5

Alkaline phosphatase (U/L)	573 ± 99	BDL: 420 ± 84 BDL + AM: 324 ± 0	BDL: 635.6 ± 197.53 BDL + AM: 419 ± 56.57	BDL: 421 ± 172.52 BDL + AM: 278 ± 171

GGT (U/L)	0 ± 0	BDL: 24.75 ± 28.93 BDL + AM: 79 ± 0	BDL: 35.8 ± 21.29 BDL + AM: 13.5 ± 19.09	BDL: 33 ± 27.4 BDL + AM: 1.86 ± 3.49^**††**^

Bilirubin (mg/dL)	0.1 ± 0	BDL: 2.73 ± 2.86 BDL + AM: 10.7 ± 5.23	BDL: 2.84 ± 1.68^∗^ BDL + AM: 2.65 ± 3.61	BDL: 4.23 ± 1.12 BDL + AM: 2.39 ± 2.50

ALT: alanine transaminase; GGT: gamma-glutamyl transpeptidase; PO: postoperative.

^∗^ *p* < 0.05 BDL versus sham.

^‡^ *p* < 0.05 BDL + AM versus sham.

^†^ *p* < 0.05 BDL versus BDL + AM.

^††^ *p* < 0.01 BDL versus BDL + AM.
